# When Strangers Become Family: The Role of Civil Society Organizations in the Care of Older People

**DOI:** 10.1093/geroni/igab046.1842

**Published:** 2021-12-17

**Authors:** Ronald Angel, Veronica Montes De Oca

**Affiliations:** 1 UT Austin, UT Austin, Texas, United States; 2 UNAM, Mexico City, Distrito Federal, Mexico

## Abstract

As in other nations, the aging of the population of Mexico presents many challenges specially in dependence. These social and political changes occur in the context of a series of interacting political, social and demographic transformations. At the end of the 20th and beginning of the 21st Centuries civil society organizations have begun to define a third sector. A growing desire of individuals to exercise more direct democracy, has accompanied the growth of identity politics and the rise of groups representing women, indigenous populations, racial and religious minorities, environmental interests, older persons, and others. These groups have changed public discourse and today give individuals greater capacity to demand their basic human and social rights. This paper reviews the impact of these changes on older people and multidimensional care.

